# Emerging innovations on exosome-based onco-therapeutics

**DOI:** 10.3389/fimmu.2022.865245

**Published:** 2022-08-31

**Authors:** Xiaofeng Dai, Yongju Ye, Fule He

**Affiliations:** ^1^ Wuxi School of Medicine, Jiangnan University, Wuxi, China; ^2^ CAPsoul Medical Biotechnology Company, Ltd., Beijing, China; ^3^ Department of Gynaecology, Lishui Hospital of Traditional Chinese Medicine, Lishui, China; ^4^ First School of Clinical Medicine, Zhejiang Chinese Medicine Museum, Zhejiang Chinese Medical University, Hangzhou, China

**Keywords:** exosome, cold atmospheric plasma, immunotherapy, cancer, therapeutics

## Abstract

Exosomes, nano-sized extracellular vesicles for intercellular communications, are gaining rapid momentum as a novel strategy for the diagnosis and therapeutics of a spectrum of diseases including cancers. Secreted by various cell sources, exosomes pertain numerous functionalities from their parental cells and have enhanced stability that enable them with many features favorable for clinical use and commercialization. This paper focuses on the possible roles of exosomes in cancer therapeutics and reviews current exosome-based innovations toward enhanced cancer management and challenges that limit their clinical translation. Importantly, this paper casts insights on how cold atmospheric plasma, an emerging anticancer strategy, may aid in innovations on exosome-based onco-therapeutics toward improved control over cancers.

## 1 Introduction

Exosomes, firstly discovered in the early 1980s, represent a class of extracellular vehicles (EVs) at a size of 30~150 nm in diameter that are secreted by all types of cells (1, 2). Exosomes were initially considered as a means for maturing reticulocytes to get rid of superfluous proteins and discard garbage (1, 2). Accumulating evidence has suggested their other roles besides eliminating unwanted molecules from parental cells such as intercellular communications, cell content exchange, immune system modulation, antigen presentation, and pathogen propagation (3–5). With our incremental understanding on exosome, its prominent functionalities under both normal and pathophysiological conditions such as lactation (6), immune homeostasis (7), neuronal signaling (8), and disease development (9, 10) have been acknowledged and taken advantages of for the purpose of theranostics.

A rising momentum has been witnessed on exosome recently, in both academia and industry. Exosome has been integrated into the field of precision medicine as it provides an extremely useful source of biomarkers for cancer diagnosis and an excellent tool for immune-therapeutics and drug delivery (7, 11). The diverse range of biofluids capable of producing exosomes makes exosome-based diagnostics minimally invasive, easy to use, and fast in detection (12, 13). By isolating a patient’s exosomes, modifying them with appropriate nucleic acids or proteins, and transfusing them back to the patient (14, 15), exosomes can be administrated in clinical practice in a similar fashion to adoptive cell therapy (ACT) that is characteristic of extreme personalization. At the same time, exosomes offer a cell-free solution. That is, they can be manufactured *ex vivo* free of cells and thus be exempted from the risks and difficulties of administering cells to patients (16).

For these beneficial traits, the era of exosomes has arrived. In academia, there had been approximately 20,000 publications on exosomes, among which three quarters have been published within the past 5 years. Clinically, at least 204 clinical trials associated with exosomes have been launched, with 114 and 74 trials related to therapeutics and diagnosis, respectively, and two diagnostic tests (i.e., Bio-Techne’s ExoDx Prostate IntelliScore Test for prostate cancers [17), Guardant360 CDx test for non-small cell lung cancers (18)] have been approved by the FDA. Commercially, at least seven partnership deals, eight large venture capital events, and two landmark acquisitions have occurred in the exosome industry during the past 5 years. Given the surging amounts of scientific publications, the rising number of clinical trials, and the swelling appetite among investors for exosome biotechnology, it is imperative to characterize the features of exosomes (derived from various sources) that enable their diversified innovative theranostic applications and, ultimately, our improved power over diseases including cancers.

Among the varied types of disorders and clinical applications where exosomes may intervene (19, 20), this review focuses on cancer therapeutics. We briefly introduce some basic information on exosomes in Section 2, categorize the primary characteristics of exosomes enabling their onco-therapeutic potential (i.e., immune modulation and cargo delivery) in Section 3, cast our insights on current and emerging innovations relevant to exosome-associated onco-therapeutic strategies in Sections 4 and 5, and focus on the status and challenges in the clinical translation of exosomes as an onco-therapeutic tool in Sections 6 and 7. Importantly, we forecast possible synergies cold atmospheric plasma (CAP), an emerging onco-therapeutic, can create with exosomes toward enhanced cancer control.

## 2 Exosome biogenesis and composition

The initial form of exosome in eukaryotic cells is early endosome which is tube-like and distributed in the peripheral part of the cytoplasm (3). When early endosome develops into late endosome, it becomes spherical, accumulates around the nucleus, and forms intraluminal vesicles inside the lumen (3). Late endosome either fuses with the lysosome toward content degradation or fuses with the cell membrane to release its intraluminal vesicles in the form of exosomes (3) (**Supplementary Figure 1**). Exosomes can be secreted either constitutively or in an inducible manner (21, 22). Tumor suppressors such as p53 can activate exosome production and secretion in response to external stimuli such as oxidative or toxic stress (22). It has been demonstrated that irradiation of human prostate cancer cells can trigger DNA damage and thus induce a p53-depenent increase in exosome secretion (23). Once exosomes are taken up by other cells, they can either release their inner content without affecting the membrane integrity or be entirely endocytosed and re-legated to clathrin-coated pits (3).

Exosomes contain many diversified cell surface molecules that allow them to participate in intercellular material exchange. The content of exosomes is complex which contains thousands of proteins and nucleic acids, as well as hundreds of lipids (24). Exosomes are enriched with proteins involved in antigen presentation such as CD1 and major histocompatibility molecules I and II (MHCI/II), co-stimulatory molecules such as CD86, adhesion molecules such as CD11b and CD54, heat shock proteins such as HSP70 and HSP90, cytoplasmic proteins such as Annexins and Rab proteins, membrane proteins such as CD55 and CD59, signal transduction proteins such as G-proteins, and protein kinases (3). Exosomes do not contain mitochondrial, nuclear, and endoplasmic proteins (25). Exosomes derived from immune cells display proteins that pertain their roles in immune responses in addition to typical exosome contents; e.g., exosomes secreted by dendritic cells (DCs) contain CD80 and CD86 for naïve CD4+ T-cell activation (26). Exosomes also contain numerous nucleic acids such as let-7, miRNA-1, miRNA-15, and miRNA-16 which play critical roles in angiogenesis, hematopoiesis, exocytosis, and tumorigenesis (25). Recent studies have reported the critical functionalities of exosome-derived long non-coding RNAs (lncRNAs) in cancer initiation and development. For instance, PTENP1 in exosomes derived from normal cells confers tumor-suppressive roles on breast cancer cells both *in vitro* and *in vivo* (27). Compared with the source cells, lipids contained in exosomes are enriched with cholesterol, sphingomyelin, glycosphingolipids, hexosylceramide, lactosylceramide, and phosphatidylserine and contain less phosphatidylcholine, phophatidylethanolamine, phosphatidylglycerol, phosphatidylinositol, and cholesteryl ester (28). Exosomes have asymmetric membrane bilayers regarding lipid decomposition (3). While sphigomyelins, sphingolipids, and most phosphatidylcholines are distributed in the outer leaflet of exosomes, all other types of lipids are mainly concentrated in the inner leaflet (28). Exosomal cargos are involved in various signalings in recipient cells and can modulate diverse biological processes such as autophagy (29) and inflammation (30).

## 3 Exosome for onco-therapeutics

### 3.1 Immunotherapy

#### 3.1.1 Exosomes of immune origin

Innate and adaptive immune responses are two major immune systems of vertebrates (31). While DCs, natural killer cells (NKs), and macrophages are essential players in the innate immunity, B and T cells are major immune cells in the adaptive immune response (31). In the adaptive immune response that is specific to vertebrates, B cells recognize foreign antigens by themselves, and T cells identify antigens with the aid of antigen-presenting cells (APCs) such as DCs, macrophages, and B cells (31) (**Supplementary Figure 2**).

Exosomes of immune cell origin have strictly defined molecular profiles that can be used for immune boosting. Exosomes derived from DCs, NK cells, and type I macrophages (M1) are known to promote cancer cell death directly or *via* presenting tumor antigens to T cells (7). Given the immune-promotive roles of exosomes derived from these immune cells, they have been proposed with profound utilities in immune-therapeutics (**Table 1**). Specifically, DCs function in immunotherapy by presenting tumor antigens to naïve T cells, and exosomes secreted from DCs contain CD80 and CD86 that are required for naïve CD4^+^ T-cell activation (26). In addition, DC-derived exosomes can overcome the limitations of DC cells in suffering from a short lifespan once activated (32, 33) by enabling their long-term storage at -80°C (34). Also, exosomes of DC origin are more efficient in antigen presentation than DCs as they have 10~100-fold more abundant MHCII molecules expressed on the surface (35) and can directly kill cancer cells by expressing peptides capable of activating NK cells (36). Besides, DC-derived exosomes are more stable than DCs in therapeutic preparation and have strictly defined molecular profiles for each patient that can be used to determine the molecular parameters for quality control (35). NK cells recognize tumor antigens primarily *via* surface-activated receptors and secrete cytotoxic molecules such as perforin and granzyme to lyse malignant cells (37). Similarly, NK cell-derived exosomes can kill cancer cells by providing FasL and perforin/granzyme, with the efficacy demonstrated in various tumors such as breast cancer (38) and neuroblastoma (39). NK-derived exosomes may take on the cytolytic activity after a short time interval and/or at low concentrations and are advantageous in being detectable in peripheral blood, diffusible into tissues, and thus having a cytolytic effect at the tumor sites (37). Thus, efforts have been devoted to construct engineered NK exosomes toward synergistic benefits. For instance, light-activatable silencing NK-derived exosomes were generated by engineering NK cells with hydrophilic small interfering RNA (siRNA) and hydrophobic photosensitizer Ce6, which can boost DC maturation and M1 polarization besides eliciting effective NK-cell cytotoxicity against tumor cells and triggering a photodynamic therapeutic effect (57). Compared with DC and NK cell-derived exosomes, relatively little has been reported on M1-derived exosomes. M1 activates the anticancer immune response by functioning as a type of APCs and producing the type I interferon IL12 and nitric oxide (58). M1-derived exosomes were proposed as promising vaccine adjuvant, since they displayed a tropism toward lymph nodes, induced Th1 cytokine release, and stimulated a strong T-cell cytotoxicity, which collectively fostered a pro-inflammatory microenvironment in the lymph nodes (40). Also, M1-derived exosomes can synergize with their encapsulated agents toward enhanced antitumor effects such as the improved efficacy of cisplatin observed in exosome-treated lung cancer cells (41).

**Table 1:** Advances and limitations of using exosomes originated from different immune cells for cancer control.

Source cell	Example contents	Impact	Advantages	Disadvantages	References
RDC	CD80, CD86.	Stimulating T-cell immunity.	Long-term storage; efficient in antigen presentation; directly kill cancer cells; good stability; easy for quality control.	NA	(26, 32–36)
NK cells	FasL, perforin, granzyme.	Directly killing cancer cells.	Effective after short time interval or at low concentrations; detectable in peripheral blood, diffusible into tissues.	NA	(37–39)
M1 macrophages	IL12, nitric oxide	Activating anticancer immune response.	Tropism toward lymph nodes; stimulating T-cell cytotoxicity.		(40, 41)
M2 macrophages	IL10, TGFβ, arginase, growth factors, angiogenic factors.	Promoting tumor growth and invasion.	NA	Circulating invasion-potentiating miRNAs in the peripheral blood.	(42)
Neutrophils	Cytokines, proteases.	Tumor-promotive; tumor-suppressive.	Loading tumor-suppressive contents from neutrophils.	Loading tumor-promotive contents from neutrophils.	(43, 44)
Treg cells	TGFβ, IL10, IL35.	Inhibiting anticancer immune response; promoting tumor angiogenesis.	NA	Inhibiting T-cell proliferation, IFN production, and T-cell cytotoxicity.	(45, 46)
Mast cells	MMP2/9, VEGF, proteases, MHCII, CD86, CD40, CD40L, ICAM-1	Tumor-promotive.	NA	Shuttling tumor-promotive molecules inherited from mast cells.	(47–49)
MDSC	S100A8, S100A9.	Tumor-promotive.	NA	Suppressing T cells, polarizing macrophages toward M2, accelerating tumor angiogenesis.	(50)
MSC	CD9, CD81, CD29, CD44, CD73.	Tumor-promotive.	NA	Blocking cytotoxicity of T cells and NK cells, recruiting macrophages; suppressing activation of T, B, NK cells; inducing Treg cells.	(51–54)
T cells	MM9, PD-1.	Tumor-promotive; tumor-suppressive.	Restoring immune surveillance.	Enhancing tumor invasion.	(55, 56)

Exosomes derived from immune cells such as type II macrophages (M2), neutrophils, T regulatory (Treg) cells, mast cells, myeloid-derived suppressor cells (MDSCs), and mesenchymal stem cells (MSCs) are tumor promotive (**Table 1**). Specifically, M2 can form a tumor-promotive immunity and are characterized by suppressed expression of MHCII and IL12 and enhanced expression of IL10, TGF*β*, arginase, growth factors, and angiogenic factors (59). M2-derived exosomes can circulate invasion-potentiating miRNAs in the peripheral blood and deliver them to cancer cells for promoted cancer cell growth and invasion, with one example being the delivery of miRNA-223 to breast cancer cells (42). Neutrophils play both tumor-promoting and tumoricidal functions in the innate immune system through the production of, e.g., cytokines and proteases (60). Neutrophil-derived exosomes convey tumor-promotive roles by loading these parental contents (43). It was reported that neutrophil-originated exosomes promoted the proliferation of lung cancer cells *via* releasing elastases, a neutrophil-derived proteinase that causes complex pathway alterations in malignant cells toward hyperactivated PI3K and AKT signaling (44). Treg cells are tumor-promotive *via* inhibiting the anticancer immune response and promoting tumor angiogenesis (61). Exosomes of Treg-cell origin can help construct an immune microenvironment favorable for tumor growth. As demonstrated using a melanoma cell model, Treg-derived exosomes inhibited T-cell proliferation, IFN production, and CD8^+^ T-cell cytotoxicity (45, 46). Mast cells are hematopoietic cells of the immune system with a detrimental impact on allergic reactions and accumulate in the tumor site to form the tumor microenvironment (TME). Factors secreted by mast cells such as matrix-degrading enzymes (MMP2, MMP9), vascular endothelial growth factor (VEGF), proteases, MHCII proteins, co-stimulatory factors (CD86, CD40, CD40L), and adhesion-related molecules (ICAM-1) are tumor-promotive (47, 48) and can be shuttled by mast cell-derived exosomes to promote carcinogenesis. For instance, exosomes of this type promoted the growth of lung adenocarcinoma cells by transferring KIT (a member of the tyrosine kinase family of growth receptors) to cancer cells, where KIT positivity was associated with short-term lung cancer survival (49). MDSCs represent a population of immature myeloid cells capable of suppressing cytotoxic T cells, polarizing macrophages toward the M2 state, and accelerating tumor angiogenesis *via* producing some soluble immunosuppressive mediators (50). MDSC-derived exosomes transferred these mediating molecules, such as S100A8/S100A9 in breast cancers (50), from sender to receiver cells to trigger M2 polarization. MSCs orchestrate the tumor immune microenvironment together with immune cells and promote tumor growth *via* blocking cytotoxic responses of T cells and NK cells and recruiting macrophages (62). Similarly, exosomes isolated from human MSCs suppressed the activation and proliferation of T cells, B cells, NK cells, and induced Tregs (51–54).

Exosomes derived from T cells play double-edged roles on cancer (**Table 1**). It was reported that exosomes of T-cell origin led to an increased invasion of melanoma and lung cancer cells *via* secreting MM9 (a marker characteristic of tumor migration) (55). However, it was lately demonstrated that T cell-derived exosomes restored immune surveillance against triple-negative breast cancer cells by secreting PD-1 that led to PD-L1 internalization and attenuated suppression on T-cell activity (56).

Besides cancer-associated traits, exosomes may convey other therapeutic efficacies due to their inherited bioactive molecules from donor cells. For instance, exosomes originating from M2 macrophages delivered parental anti-inflammatory cytokines that accelerated wound healing (63); exosomes of MSC origin stimulated angiogenesis-related factors that modulated immunity and promoted tissue regeneration (64).

#### 3.1.2 Exosomes of cancer origin

The roles of exosomes derived from cancer cells in immune response are controversial. For instance, while acute lymphoblastic leukemia-derived exosomes inhibited the cytotoxicity of NK cells by enhancing TGFβ signaling (65), leukemia-derived exosomes activated the anticancer immune response by downregulating TGFβ1 expression (66). Also, exosomes derived from head and neck squamous cell carcinoma patients carried inhibitory factors that are immune suppressive (67). Tumor cell-derived exosomes, once functionalized, can act as an antitumor immunotherapy. For instance, hepatocellular carcinoma cell-derived exosomes painted with the functional domain of HMGN1 boosted the ability of DCs in activating T cells toward long-lasting anticancer immunity both *in vitro* and *in vivo* (68).

Exosomes derived from cancer-associated fibroblasts (CAFs) and endothelial cells are mostly tumor-promotive as they typically transfer “signals” to tumor cells and promote tumor progression. For instance, CAF-derived exosomes were obligated to induce metabolic reprogramming toward enhanced tumor growth and metastasis (69). Exosomes derived from brain microvascular endothelial cells were found capable of enhancing the survival of small-cell lung cancer cells metastasized to the brain by delivering and enhancing the expression of S100A16 using an *in vitro* cell coculture system (70).

#### 3.1.3 Exosomes of food origin

Exosomes derived from food such as milk and edible plants may inherit some features from their parental sources and thus *per se* be preventive against some undesirable immune responses. For instance, exosomes derived from milk contain immune-related miRNAs that showed excellent therapeutic efficacy against inflammatory bowel diseases *in vivo* (71); grape-derived exosomes modulated intestinal homeostasis and were protective against inflammatory bowel diseases (72) such as dextran sulfate sodium-induced colitis (73); and ginger-originated exosomes inhibited the activation of the NLRP3 inflammasome, a key innate immune response regulator typically activated in Alzheimer’s diseases and type II diabetes (74).

### 3.2 Drug delivery

Nano-vehicles for drug delivery can be synthesized or biologically derived at the scale of nanometers that has the physiochemical properties for targeted delivery of agents against cancerous cells. Various nanoparticles have been employed for this purpose including metallic nanoparticles (75), polymeric nanoparticles (76–78), lipid-based carriers such as liposomes and micelles (79), and viral nano-vehicles (80). Metallic nano-carriers suffer from metallic toxicity as a result of particle accumulation in vital organs and difficulty in entire clearance (75). Polymeric nanoparticles are primarily limited by their low yield and biodegradability (78). Lipid-based nano-carriers, although having little concern on toxicity and yield, have biocompatibility issues such as the cause of mucositis (81). Although viral particles can be produced in a large amount at a relatively low cost without biocompatibility issues, they raise safety concerns due to potential spontaneous mutations (82).

Exosomes offer an excellent solution to overcome these aforementioned shortcomings in drug delivery. Compared with artificial or conventional nanoparticles, exosomes are advantageous in easier blood circulation clearance (83), large capacity for *ex vivo* expansion (11), and biocompatibility due to their endogenous origin (84). Also, exosomes have high cellular uptake due to the existence of membrane proteins such as integrin (85), tetraspanin (CD9, CD63, CD81) (86), and fibronectin (87), are flexible in surface modification (88, 89), and can evade the immune system toward prolonged body circulation time (90). Importantly, exosomes can overcome biological barriers such as the blood–brain barrier (BBB) (88) and lung clearance (91, 92) which make their roles in drug delivery more promising.

#### 3.2.1 Origins of exosomes for drug delivery

**Figure 1:**
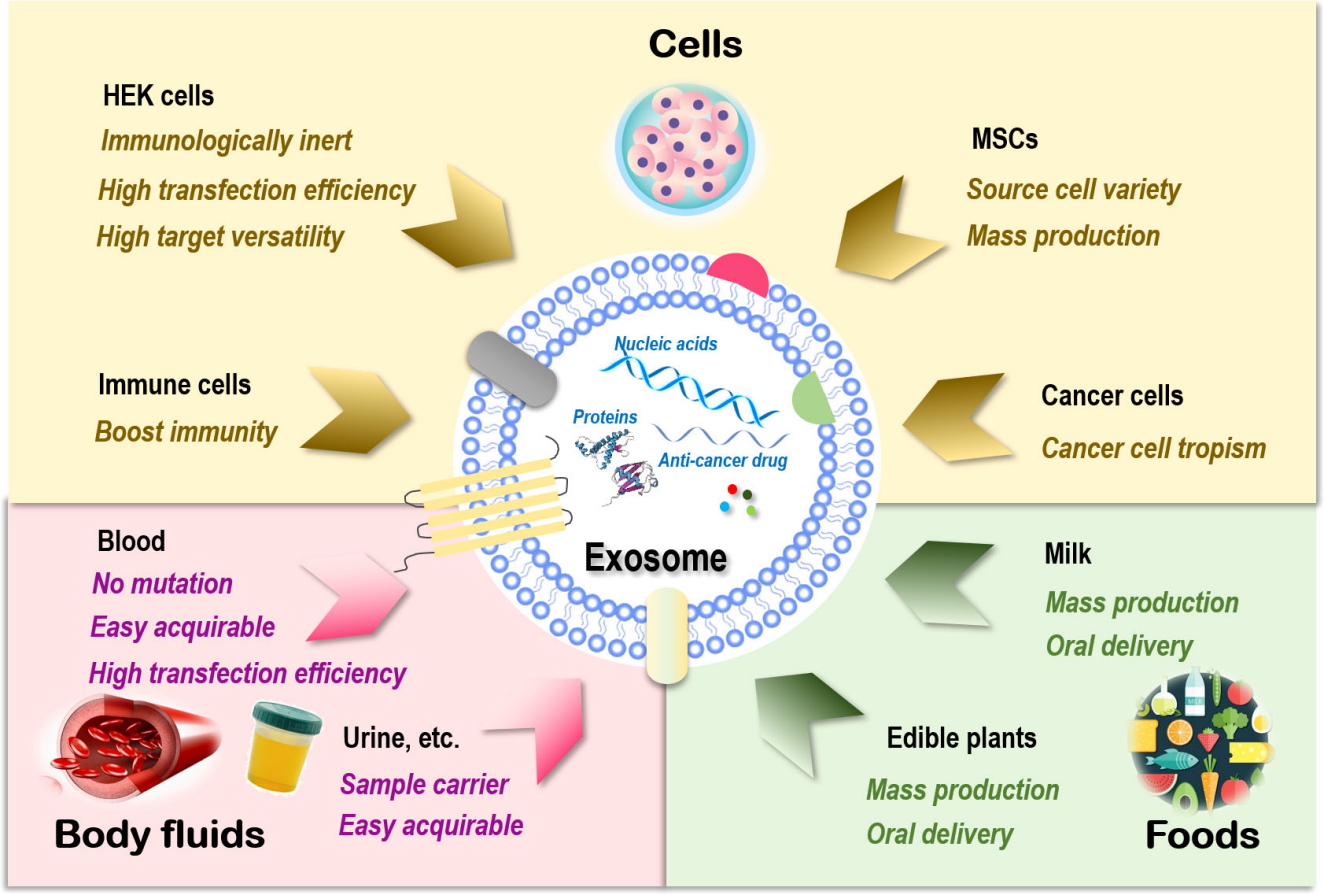
Sources of exosomes and their therapeutic advantages for drug delivery. Exosomes can be generated from almost all types of cells. Those are relevant to therapeutics fall into three main categories, i.e., cells, body fluids, and food. There are four major cell sources for exosome generation, i.e., human embryonic kidney (HEK) cells, mesenchymal stem cells (MSCs), immune cells, and cancer cells. Exosomes of HEK cell origin are immunologically inert without safety concerns, have high transfection efficiency, and can deliver drugs to various target tissues. Exosomes derived from MSCs can be easily obtained from a variety of human tissues and expanded ex vivo in large scale. Exosomes originated from immune cells can enhance the anticancer efficacy of its entrapped drugs by boosting the immunity. Exosomes derived from cancer cells have the tropism toward their parental cells and thus can be used as Trojan horses to target these malignant cells. Exosomes derived from blood have a low risk of unexpected mutations, are easily acquirable, and have higher transfection efficiency. Exosomes isolated from other body fluids such as urine, saliva, and amniotic fluids can be used as parental cell sample carriers for disease diagnosis. Exosomes derived from food such as milk and edible plants are stable under acidic conditions that can be orally delivered and massively produced.

Sources of exosomes that can be used as drug carriers include cells (93), body fluids such as blood (94), and food such as milk (95). Exosomes of cell origin can be derived from, e.g., human embryonic kidney (HEK) cells, stem cells, immune cells, and cancer cells (**Figure 1**).

HEK cells, extensively used as an expression tool for recombinant proteins, can also be employed for exosome production, and these exosomes are immunologically inert without safety concerns (96), are highly efficient in transfection, and have membrane resemblances to various human tissues that make it possible to deliver drugs to various target tissues (97, 98). In addition, HEK-derived exosomes can deliver the natural forms of membrane proteins to target cells that are fully functional, resulting in improved tumor penetration and antitumor efficacy. For example, expressing native PH20 hyaluronidase on exosome surface remarkably enhanced the anticancer efficacy of exosomes carrying doxorubicin (Dox) *in vivo* by degrading hyaluronan in the tumor extracellular matrix (99). Yet, the yield of HEK-derived exosomes was lower than that of exosomes originated from body fluid and food (100).

Exosomes derived from stem cells such as MSCs can be easily obtained and expanded *ex vivo* (93). MSC represents an ideal source for exosome preparation as cells of this kind can be obtained from a variety of human tissues. Large-scale production of good manufacturing practice (GMP)-grade exosomes for clinical use has been made available from bone marrow-derived MSCs, where the quantities were threefold those of exosomes obtained from human foreskin fibroblasts (101). MSC-derived exosomes delivering siRNAs that target *Kras*, namely, “MSCs siKras Exo,” effectively resolved tumors and halted tumor metastasis *in vivo* (102).

Exosomes derived from immune cells can enhance the anticancer efficacy of the entrapped drugs. For instance, the effectiveness of paclitaxel in killing breast cancer cells was substantially enhanced once encapsulated in NK-derived exosomes (103). Another study used macrophage-derived exosomes to load paclitaxel, where an aminoethylanisamide-polyethylene glycol (AA-PEG) vector moiety was incorporated to target the sigma receptor overexpressed in lung cancer cells (104). Similarly, a macrophage-derived exosome-coated poly(lactic-co-glycolic acid) nano-vehicle was established for targeted chemotherapy against triple-negative breast cancers, where the exosome surface was modified with a peptide against c-Met toward enhanced targetability (105).

Exosomes derived from cancer cells have tropism toward their parental cells and thus can be used as Trojan horses to target these malignant cells (105–107). For example, the anti-inflammatory activity of curcumin was improved when encapsulated in breast cancer cell-derived exosomes as a result of the innocent bystander or off-target effect (108). However, in-depth investigations on the metastatic roles of these exosomes are needed before they can be safely used for anticancer drug delivery.

Blood-derived exosomes have a relatively low risk of unexpected *in vitro* mutations during cell culture due to the large percentage of red blood cells (enucleated) contained in their source materials. In addition, exosomes derived from blood are easily acquirable (i.e., from blood banks or patients) and have higher transfection efficiency. These advantages have made exosomes of this kind ideal delivery vehicles for nucleic acid-based therapeutics (94). Exosomes isolated from other types of body fluids such as blood plasma, urine, saliva, and amniotic fluids can be used as parental cell sample carriers for disease diagnosis (109–112).

Exosomes derived from food are excellent sources for effective delivery of encapsulated therapeutic molecules as, e.g., milk is stable under acidic conditions that can be orally delivered (113, 114) and massively produced (92). Orally administered exosomes loaded with paclitaxel have been shown capable of substantially inhibiting tumor growth *in vivo* without obvious side effects (115). In addition, milk exosomes can be functionalized toward enhanced stability and biocompatibility. For instance, polyethylene glycol (PEG)-engineered milk exosomes showed around 3.2-fold increased mucus permeability than their unmodified peers (116).

#### 3.2.2 Cargos of exosomes for therapeutics

Cargos imbedded in exosomes as cancer therapies can be in various forms including nucleic acids, proteins, and anticancer agents.

Nucleic acids such as miRNAs, siRNAs, mRNAs, lncRNAs, and circular RNAs can all be delivered by exosomes. Desired RNAs can be loaded into exosomes *via* overexpressing candidate RNAs in parental cells. For example, exosomes encapsulating miRNA-122 can be obtained by transfecting plasmids expressing miRNA-122 into adipose tissue-derived MSCs (117). Alternatively, nucleic acids can be transferred into exosomes through electroporation such as in the case of loading antisense miRNA-21 into exosomes (118). As endeavors to achieve therapeutic goals, exosome-delivered miRNA-375-3p mimic significantly suppressed the EMT process of colon cancer cells (119), and exosome-mediated delivery of the miRNA-142-3p inhibitor suppressed breast cancer tumorigenicity both *in vitro* and *in vivo* (120). Breast cancer cell-derived exosomes were used to deliver *S100A4* siRNA that led to significantly reduced postoperative breast cancer metastasis (121), and exosomes loaded with *SCD-1* siRNA significantly promoted anaplastic thyroid carcinoma cell apoptosis by enhancing its intracellular ROS level (122). Exosomes loaded with mRNAs encoding SARS-CoV-2 spike and nucleocapsid proteins triggered long-lasting immune response both *in vitro* and *in vivo* (123), and exosome-mediated delivery of *IL10* mRNA effectively alleviated atherosclerosis (124). Exosome-transmitted lncRNA SENP3-EIF4A1 suppressed the progression of hepatocellular cancer cells (125), and exosome-delivered lncRNA PTENP1 inhibited the development of bladder cancer cells (27). Exosome-carried circular RNA hsa_circ_0051443 suppressed hepatocellular cancer progression (105).

Similar to nucleic acids, proteins can be encapsulated into exosomes *via* either genetic engineering of donor cells or physical loading such as electroporation and detergent-based approaches. For instance, surviving-T34A was overexpressed in exosomes derived from melanoma cells through plasmid transfection, leading to enhanced gemcitabine sensitivity and significant apoptosis of pancreatic adenocarcinoma cells (126). Tyrosinase-related protein-2 (TRP2) was loaded into exosomes of the serum origin through the use of the detergent saponin or electroporation, which were internalized into macrophages and DCs to effectively stimulate the adaptive immune response (127). Another interesting attempt for exosome protein encapsulation relying on genetic engineering is to load tumor-specific antigens or immune stimulants on exosome surface for vaccination. For instance, by infecting a mouse DC cell line with lentiviruses encoding the α-fetoprotein (*AFP*) gene, DC-derived exosomes expressing AFP induced a robust immune response *in vivo* that led to suppressed tumor growth and prolonged animal survival (128). Using a similar strategy, exosomes expressing signal-regulatory protein alpha (SIRPα) on the membrane surface were constructed that can avoid failed immune surveillance *via* blocking the recognition of CD47 (expressed by tumor cells) by immune cells, leading to enhanced phagocytic ability of macrophages and inhibited growth of cancer cells *in vivo* (9, 10).

Anticancer drugs can be directly loaded into exosomes as onco-therapeutics. Approaches enabling drug loading primarily include incubation, sonication, and electroporation, with the most commonly reported drugs for exosome encapsulation being Dox and paclitaxel (PTX) (100, 129). Compared with free Dox or Dox loaded by liposomes, Dox delivered by exosomes showed a superior anticancer efficacy against colon cancers *in vivo* (130); this is attributable to the optimized endocytosis as determined by the cholesterol and the phospholipid composition of the exosome membrane surface (131). MSC exosome-delivered Dox enhanced the cellular uptake and anticancer effect of Dox against osteosarcoma due to the tropism of MSC toward tumor tissues (132). Another advantage of exosome-delivered Dox is its enhanced safety, since exosome can prevent its encapsulated agents from being delivered to myocardial endothelial cells that may lead to cardiotoxicity (133).

## 4 Exosome innovations for improved onco-therapeutics

### 4.1 Exosome mimetics

Although red blood cell-derived exosomes are featured with high yield, most studies obtain exosomes from MSC or immortalized cells. Thus, how to effectively enhance exosome yield still imposes one major obstacle limiting the development of exosome-based therapeutics.

One innovation to resolve this issue is the development of nano-sized exosome mimetics (also called “nanovesicles” or “hybrid exosomes”). Exosome mimetics are artificial delivery vehicles mimicking exosomes, which are advantageous in high yield and flexibility in content genetic engineering as compared with exosomes. These features enable them with higher pharmaceutical acceptability due to their more effective and safer delivery manner and well-characterized content (134). However, incorporating multiple proteins on the membranes of exosome mimetics is complex and time-consuming, and the functionalities of the incorporated proteins need to be validated (134).

Technologies enabling the delivery of nucleic acids through exosome mimics have been established. One strategy is to produce size-controllable exosome mimics *via* serial extrusion of non-tumorigenic MCF10A cells through filters of various pore sizes followed by encapsulating siRNA using electroporation (135). The yield of exosome mimics increased approximately 150-fold as compared with that of exosomes without sacrificing the efficiency and safety (135). The roles of MSC-derived exosome mimetics as alternative vesicles of exosomes for drug delivery have been evaluated, where PTX and breast cancer cells were used as the drug and tumor models, respectively (136). The results showed that MSC-derived exosome mimetics could be easily isolated using simple protocols, and drug-loaded mimetics could effectively kill breast cancer cells both *in vitro* and *in vivo* (136). Besides successes reported on MSC-derived exosome mimetics, mimetics of immune cells such as macrophages and NK cells were proven effective for treating various types of cancers (137, 138).

### 4.2 Exosome surface modification

Surface modification of exosomes can impart additional functionalities to exosomes such as ① sensitizing TME and stimulating immune response, ② improving tumor targetability and retention, and ③ *in vivo* imaging and trafficking (139).

The purposes of the first two categories are meant for optimized therapeutics and often involve genetic engineering. For instance, by modifying exosomes with folic acids, Feng et al. constructed a novel exosome-based drug delivery system, namely, Exos-PH20-PA, using genetic engineering and self-assembly techniques, and the modified exosomes effectively polarized macrophages from the M2 to M1 phenotype toward an immune-supportive state (140). Activated T cell-derived exosomes were shown to express PD-1 that attenuated PD-L1-triggered immune dysfunction in triple-negative breast cancers, suggestive of the feasibility of attenuating the suppressive TME by modifying the exosome surface with inhibitory immune checkpoint receptors (56). Toward improved tumor targetability, IL12 was displayed on exosome surface *via* fusion with the exosome surface protein PTGFRN, and the resultant exosome (exoIL12) conveyed tumor-restricted pharmacology that led to prolonged tumor retention and immune memory (141).

The purposes of the last category are designed for the *in vitro* or *in vivo* investigation of exosome features such as biodistribution, uptake, and mechanism that typically involve fluorescence labeling. For example, bone marrow MSC-derived exosomes were labeled by DiR dye followed by intraperitoneal administration using pancreatic tumor-bearing C57BL/6 mice to study *in vivo* exosome biodistribution, which revealed specific exosome retention in tumor cells (142). MDA-MB-231 breast cancer cell-derived exosomes were cultured in the hypoxic environment induced by deferoxamine (DFO) to generate hypoxic exosomes and were subjected to DiO (a fluorescent lipophilic tracer) labeling to study the uptake of hypoxic exosomes; the results showed ~97% vs. ~73.1% uptake of hypoxic vs. normal exosomes by hypoxic cells, suggesting the affinity of hypoxic cells toward hypoxic exosomes (143). Exosomes of bovine milk origin were labeled by PKH67 and incubated with H1299 lung cancer cells together with endocytosis inhibitor cytochalasin D to study the endocytosis pathway, and the results showed that cytochalasin D reduced endocytosis by 21% (144).

### 4.3 Exosome-based therapeutic synergies

Given that exosomes can function as both immuno-therapeutics and drug carriers, as well as their flexibilities in surface modification that allow for various *de novo* and improved functionalities, synergies have been sought for through exploring the varied combinatorial possibilities.

Attempt integrating the roles of exosomes in immunotherapies and drug delivery on cancer therapeutics represents an interesting and promising field trend. In other words, exosomes, especially those derived from immune cells, can be considered as both immune-modulators and drug vehicles at the same time, and by modulating exosome content, we can achieve various synergies such as improved immunotherapeutic efficacy and dual targeting. For instance, NK-derived exosomes carrying the tumor-suppressive miRNA-186 exhibited cytotoxicity against *MYCN*-amplified neuroblastoma cells, where miRNA-186 inhibited *MYCN* and exempted cancer cells from TGFβ1-dependent immune escape (39).

Lots of successes have been reported taking advantage of exosome surface modification and drug encapsulation. That is, one can achieve synergies by editing the surface of exosomes toward enabled functionalities such as improved tumor targetability and utilizing their drug delivery role. For example, expressing PH20 hyaluronidase on exosomes derived from HEK293PT cells enhanced the anticancer efficacy of Dox by degrading hyaluronan in TME when PH20 and Dox were co-delivered using exosomes *in vivo* (99).

Conventional dual-targeting strategies have been actualized using exosomes as the effective drug encapsulator and delivery vehicle. This is actually the most straightforward way of creating synergies where multiple drugs can be harmonically mixed and encapsulated into exosomes toward improved therapeutics. For instance, a cocktail therapy was established by combining a natural polymer hyaluronic acid-based hydrogel, engineered endothelial cell-derived exosomes (EC-Exos^miR-26a-5p^), and APY29 (an IRE-1α inhibitor), which could simultaneously regulate osteoblast and M1/M2 macrophage balance (145); a combinatorial strategy encapsulating TGFβR1 kinase inhibitor and TLR7/8 agonist in exosomes was shown effective in halting tumor growth *in vivo* and proposed as a novel therapeutic strategy against melanoma and prostate cancer (146).

## 5 Aid of CAP in exosome innovations for improved onco-therapeutics

Following successive innovations in creating synergies between exosomes and various drugs and exosome-modulating approaches, relatively little has been focused on the potential aid of emerging technologies in exosome innovations. Here we introduce an emerging anticancer tool, CAP, and its possible synergies with exosomes toward improved onco-therapeutics.

CAP, a fourth state of matter that relies on reactive species toward selective control over malignant cells for death (147–151), has become an emerging onco-therapeutic tool with great translational potential (152, 153). It is a cocktail of multiple reactive oxygen and nitrogen species (RONS) such as short-lived species singlet oxygen (O), hydroxyl radical (OH·), superoxide (O^2−^), and nitric oxide (NO·), and long-lived species hydrogen peroxide (H_2_O_2_), ozone (O_3_), anionic (OONO^−^), and protonated (ONOOH) forms of peroxynitrite. These species, by themselves or their interactions, interact with the surface of cancer cells and generate a series of intracellular signalings that selectively arrest cancer cells at various types of death states (such as immunogenic cell death (ICD) (154), apoptosis (150), cell-cycle arrest (147), autophagy (155), ferroptosis (156)) by perturbating their redox homeostasis. Such a selectivity not only attributes to the higher basal level of cells under the malignant state as compared with their healthy peers but also is associated with the membrane features of cancer cells. The latter includes, e.g., a high expression of aquaporins on cell surface that is associated with increased H_2_O_2_ uptake (157) and a high local concentration of catalysis on the surface that determines the specific response of tumor cells to self-destruction as a result of secondary singlet oxygen generation (158).

We identify three possible synergies that CAP may create with exosomes. That is, CAP may synergize with exosome toward enhanced immuno-modulation, function as the cargo of exosomes, and boost exosome production.

### 5.1 CAP for enhanced sensitivity to exosome-triggered immunity

**Figure 2:**
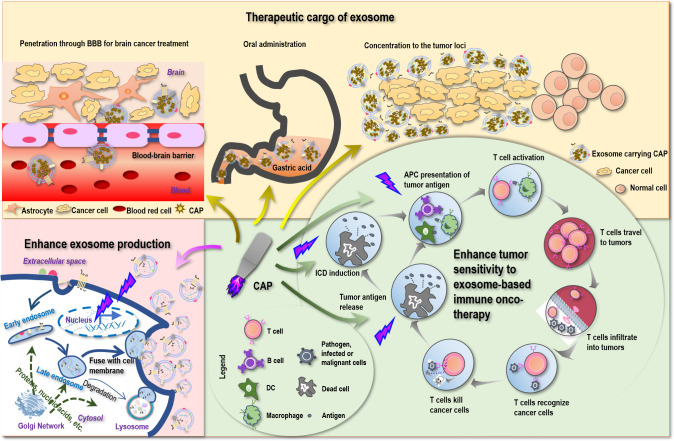
Possible scenarios where CAP creates synergies with exosomes toward conceptual and technological onco-therapeutic innovations. First, CAP can promote tumor antigen release, stimulate immunogenic cell death (ICD), enhance tumor antigen presentation by APC cells, and thus can enhance the sensitivity of tumor cells to exosome-based immune onco-therapies. Second, CAP can function as an onco-therapeutic and the cargo of exosomes to treat brain cancers by breaking the blood–brain barrier (BBB), to enable oral intake as a result of increased tolerance to gastric acidity, and to achieve enhanced drug utility by being concentrated to the tumor loci. Third, CAP can stimulate the expression of p53 that ultimately leads to enhanced exosome production.

Despite the various reports on the sensitizing role of CAP to chemotherapies (159, 160), little has been examined on the potential synergies between CAP and immunotherapies. Yet, as CAP can trigger ICD (161) that transforms non-immunogenic cells to the immunogenic state *via* promoting the release of tumor antigens, it is plausible to believe that CAP may enhance the antitumor immunity if applied together with immunotherapies including those based on exosomes (**Figure 2**). Besides, ROS are known to promote the expression of MHCI for improved macrophage-mediated tumor antigen presentation toward enhanced T-cell adaptive immune response (162) (**Figure 2**), providing additional support for the theoretical basis of possible synergies between CAP and exosomes that warrants in-depth explorations.

### 5.2 CAP as an emerging therapeutic cargo

Besides the selectivity of CAP against various types of cancer cells demonstrated *in vitro* and *in vivo (* 147–151, 163, 164), intensive efforts have also been devoted to translating CAP into clinics as an onco-therapeutic modality. The first clinical success was reported in 2018 where the life of a 75-year-old late-stage pancreatic cancer patient was secured (165). The first clinical trial using CAP as a cancer therapy was issued on 30 July 2019 and completed on 14 April 2021 in USA (NCT04267575). In this trial, 20 stage IV solid cancer patients (including breast cancer, prostate cancer, pancreatic cancer, lung cancer, ovarian cancer, fallopian tube cancer, colon cancer, liver cancer, renal cell cancer, rectal cancer, small intestinal cancer, gastric cancer) were recruited, out of whom 17 patients were still alive by the completion of this study (166). These studies suggested CAP as a safe and effective novel modality against malignant cancers. Despite these successful stories, the clinical translation of CAP is hindered by several shortcomings of CAP such as limited penetration in-depth and temporary lifespan of short-lived reactive species.

Being a nano-sized endogenous traveling vehicle with many unique benefits such as BBB transverse ability, biocompatibility, tissue-specific targeting, and cargo stability protection, exosome is ideal for CAP delivery. Since CAP can be prepared in the form of liquid, namely, plasma-activated medium (PAM), it can be made as the cargo of exosomes, alone or mixed with hyaluronic acid (167) for enhanced stability or mixed with hydrogel (168) for extended release. Although CAP is mild and selective against cancer cells without affecting their healthy peers (169), targeted delivery can help concentrate CAP in the tumor loci toward enhanced “drug” utility (**Figure 2**).

The most promising is that by imbedding PAM within exosomes, it is possible to pass PAM through BBB and treat brain diseases that currently lack an effective treatment approach with little side effect. Indeed, by loading superparamagnetic iron oxide nanoparticles (SPIONs) and curcumin into exosomes and conjugating the membrane of exosomes with neuropilin-1-targeted peptide (RGE), glioma-targeting exosomes were obtained that achieved simultaneous cancer diagnosis and therapeutics (170). MSC-originated exosomes were shown as a promising approach for treating posttraumatic brain injury since, among others, these exosomes were capable of crossing over BBB, feasible for long-term storage, non-tumorigenic, non-immunogenic, and microvascular embolism non-inducive (8). Also promising is the potential to deliver PAM in the form of capsules with the aid of exosomes that can tolerate gastric acidity and enable oral drug intake (**Figure 2**), which represents an interesting research topic with strong clinical impact and translational potential.

### 5.3 CAP for inducible exosome secretion

Exosomes can be produced either constitutively or in an inducible fashion on stress (22, 23). It is known that p53, a critical player for genome stability maintenance and DNA damage repair, can be activated on oxidative stress and trigger DNA damage response that ultimately leads to enhanced exosome secretion (23). CAP, a redox modulator, was reported capable of modulating p53 in keratinocytes (171) and activating p53 pathway-related genes in cancer cells (172). These collectively suggested the possible role of CAP in promoting exosome generation (**Figure 2**), making the massive production and utilization of exosomes of various origins possible and encouraging.

## 6 Clinical efforts using exosomes as an onco-therapeutic tool

Clinical trials using exosome as an onco-therapeutic approach can be dated back to almost 20 years ago when two phase I clinical trials using immature DC-derived exosomes for treating melanoma and lung cancer were launched (14, 15). In the first trial, exosomes originated from DCs were pulsed with melanoma-associated antigen (MAGE) and inoculated to 15 stage III/IV melanoma patients, where approximately 62% patients exhibited an enhanced NK-cell activity with no major toxicity reported 2 weeks after immunization (14). In the second trial, DC-derived exosomes were loaded with HLA-restricted MAGEs, followed by back infusion into patients carrying HLA A2+ non-small cell lung cancers (NSCLCs), where one-third of the patients showed MAGE-specific T-cell responses and, among the four analyzed patients, half presented an increased NK-cell activity after a 1-month weekly treatment (15). It has been proposed that mature DC-derived exosomes can induce more potent T-cell priming than those derived from immature DCs (173). A non-randomized phase I/II clinical trial achieved antigen-specific T-cell responses among seven esophageal cancer patients using mature DC-derived exosomes pulsed with SART1 (a biomarker of squamous cell esophagus carcinoma) (174). However, the use of mature DC for exosome generation is not the sole golden standard for activating an antigen-specific T-cell response that also relies on many other factors. For instance, a phase II clinical trial documented the use of exosomes derived from mature DCs in treating patients carrying NSCLCs in 2016. In this trial, mature DC exosomes loaded with MHC class I and II-restricted cancer antigens and IFN-γ were administrated to 22 patients. The results showed elevated NK-cell activities without obvious toxicity; yet, unlike what was expected, no cancer-specific T-cell immune response was boosted (175). This may be attributable to the suppressive role of PD-1 on T-cell activity, the expression of which from exosomes was elevated by IFN-γ. Other studies documented the indispensable role of CD4+ T and B cells in activating cytotoxic T cells by DC-derived exosomes (176).

Besides DC-derived exosomes, clinical efforts in cancer treatment have also been devoted to utilize exosomes originating from other cell sources. One phase I clinical trial used ascites-derived exosomes together with granulocyte-macrophage colony-stimulating factor as a combined therapeutic for advanced colorectal cancers. After a 4-week treatment administration, colorectal cancer patients demonstrated a strong cytotoxic T-cell response against cancer cells carrying the carcinoembryonic antigen (a biomarker of colorectal cancers) (177).

Ongoing efforts have been made to translate exosomes into clinical use for drug delivery. For example, Codiak BioSciences, being one of the major biotech start-up companies dedicated for exosome therapeutic development, constructed an engineered exosome for pancreatic cancer treatment by delivering siRNAs targeting the KRAS (G12D) mutation (iExosomes) (142), for which they have obtained the investigational new drug (IND) approval for conducting the phase IA/B clinical trial from US FDA (NCT03608631).

## 7 Challenges limiting the use of exosomes as therapeutic tools

**Figure 3:**
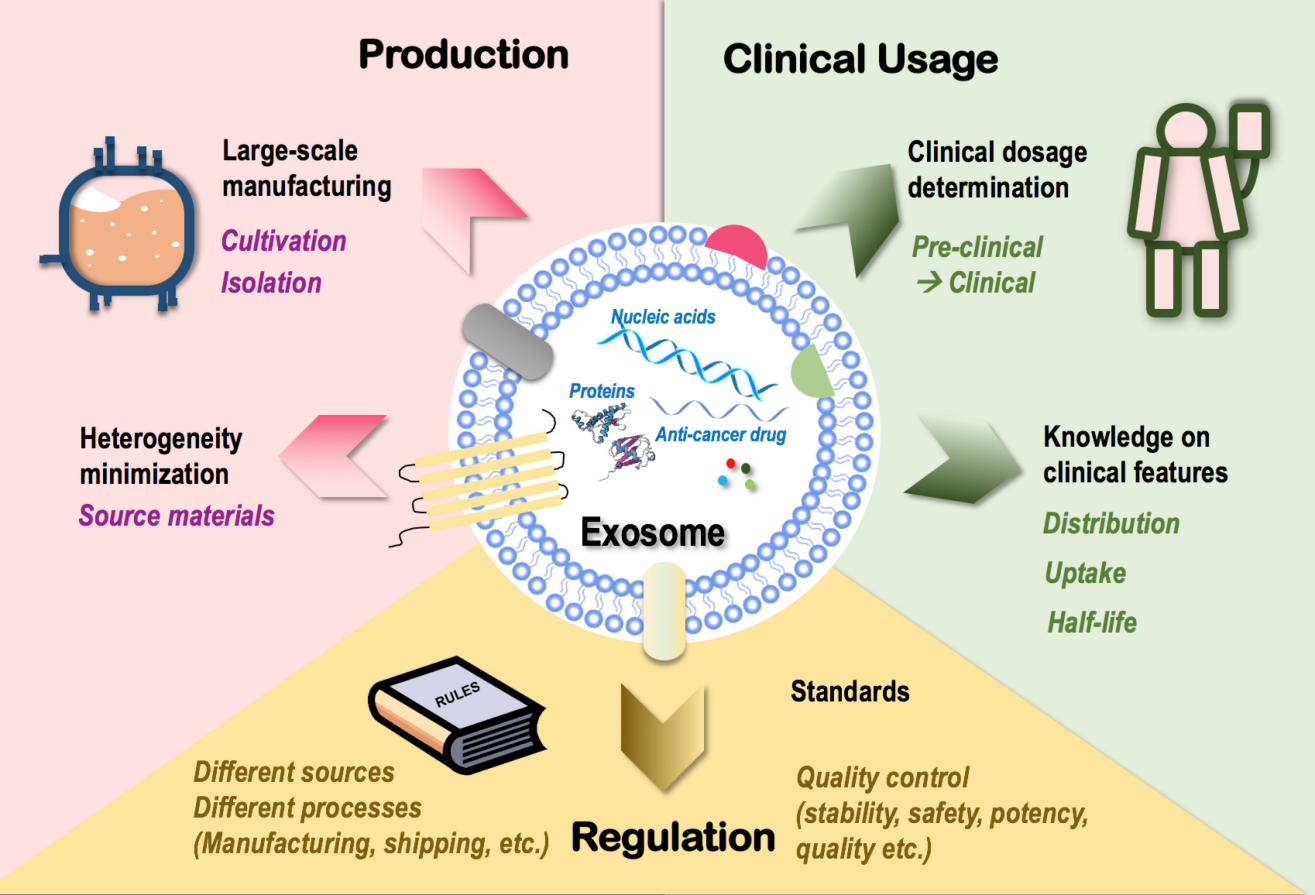
Challenges limiting the clinical translation of exosomes as onco-therapeutics. Challenges limiting the clinical translation of exosomes fall into three categories, i.e., exosome production, clinical usage, and regulation. In “production,” techniques that enable large-scale exosome manufacturing such as source cell cultivation and exosome isolation, and techniques minimizing exosome heterogeneity such as control over the heterogeneity of source materials are limiting factors. In “clinical usage,” knowledge on exosome dosage for clinical use and clinical features of exosomes such as distribution, cell uptake, and half-life are limiting factors. In “regulation,” lack of a set of industrial standards feasible for the manufacturing and shipping processes of exosomes derived from different sources and lack of quality control guidelines over exosome stability, safety, potency, and quality are limiting factors.

The many clinically favorable features superimposed on exosomes such as the immune-boosting and molecule-carrying roles have provided us with a unique option to target tumors using cell-free cancer vaccination (7) capable of synergizing with various onco-therapeutic cargoes. This has led to an industrial zest toward its clinical translation that, however, is currently limited by the following therapeutic hurdles (**Figure 3**).

Large-scale production is essential for the wide clinical application of novel techniques including exosomes. Cultivation and isolation are critical steps in exosome manufacturing. Conventional cell culture has been traditionally used for cultivating source cells of exosomes. The 3D culturing technique (through the use of bioreactors) has been established to maximize the cell culture surface area toward enhanced exosome yield (178, 179) which, however, is not necessarily cost-effective as more media and more frequent passages are needed for bioreactor-based cell culturing. Ultracentrifugation represents a dominant approach for exosome isolation that suffers from quantity loss due to exosome heterogeneity and from biological contamination (180).

How to minimize the heterogeneity of exosomes represents another important problem challenging large-scale exosome production. Among all possible influential factors, the heterogeneity of parental cells (even of the same cell type) imposes the leading effort that may substantially affect the quality, consistency, and functionality of exosomes produced. For instance, exosomes released from the muscle cells of aged mice are prone to induce inflammation and accelerate aging due to the enrichment of miRNA-34a than those derived from young mice (20, 181). Thus, a careful assessment on potential therapeutic indexes of source materials with critical impacts on the functionalities of derived exosomes such as age should be considered as an important guideline for large-scale exosome manufacturing.

Besides issues relevant to exosome mass production, there also exist unresolved problems on how to translate preclinical experiences into clinics. First and foremost is how to determine exosome dosage under different therapeutic scenarios, where experiences gained using cells or animal models need to be extrapolated into the clinical level. Other clinical features such as exosome distribution, uptake, and half-life should be carefully investigated to avoid off-target tissue accumulation prior to clinical administration (182).

Also worth mentioning is the limitations of exosomes in stimulating the immune system. Similar with canonical agents, exogenous exosomes cannot produce long-term consecutive stimulation to the immune system and may lose the immune-stimulatory efficacy if running exhausted. However, it is not feasible to inject overdosing exosomes to the human body as an overactivated immune system may lead to undesirable clinical outcomes such as cytokine storm that, sometimes, can be life-threatening. Thus, achieving extended exosome release by synergizing with materials such as hydrogel may represent a promising solution and a possible future research direction. Another remaining issue is how to determine the dose and frequency of repeated exosome administration as well as the dosage regime that may differ among patients, without which it may be difficult to achieve an optimal or expected therapeutic response. Thereby, reinforcing our control over the choice of exosome dosage under each treatment scenario represents another major obstacle awaiting to be resolved.

Lastly, lack of industrial standards limits exosome commercialization. Exosomes derived from different sources have different features and thus require different manufacturing protocols and quality control standards. Besides, the stability, safety, potency, and quality of exosomes should all be carefully controlled throughout the manufacturing process and under the shipping conditions. Thus, a set of standards systematically covering all these scenarios is urgently needed for standardized exosome manufacturing that requires collective advances in technologies and government/industrial regulations.

## 8 Conclusion

Being homogeneous EVs secreted by various types of cells for intercellular communication, exosomes carry information from host cells that can be utilized for diagnosis and, importantly, as immuno-therapeutics due to inherited features from their immune cell origin (for exosomes derived from some immune cells). On the other hand, exosomes are ideal natural nano-carriers for drug delivery due to their small sizes that allow them to penetrate through BBB, flexibilities in surface modulation that allow tissue-specific targeting, endogenous origins that enable their biocompatibilities, natural expression of surface receptors that allow their easy communication with target cells, and bilayer membrane structures that protect their imbedded cargos from, e.g., gastric acidity.

CAP, being an emerging onco-therapeutic approach and redox modulator, can aid in exosome-based onco-therapeutics by sensitizing exosome-based immunotherapies, being the cargo of exosomes for effective malignant cell removal with little side effect, and functioning as a controllable inducer for massive exosome production.

We forecast the emerging and wide application of exosomes in onco-therapeutics, and the prominent role of CAP in availing this process toward, hopefully, eventual malignant tumor eradication. Our insights not only categorize the unique traits of exosomes favorable for disease management (especially for treating brain cancers) but also identify a novel opportunity for cancer therapeutics through a combined use of exosomes and CAP.

## Author contributions

XD conceived the study, conducted the literature searching, drafted the manuscript, and prepared the figures. YY contributed in manuscript revision. FH provided the financial support. All authors contributed to the article and approved the submitted version.

## Funding

This work was supported by the National Natural Science Foundation of China (Grant No. 81972789), Fundamental Research Funds for the Central Universities (Grant No. JUSRP22011), and Technology Development Funding of Wuxi (Grant No. WX18IVJN017). The funding bodies played no role in the design of the study and collection, analysis, and interpretation of data and in writing the manuscript.

## Conflict of interest

Author XD was employed by CAPsoul Medical Biotechnology Company, Ltd.

The remaining authors declare that the research was conducted in the absence of any commercial or financial relationships that could be construed as a potential conflict of interest.

## Publisher’s note

All claims expressed in this article are solely those of the authors and do not necessarily represent those of their affiliated organizations, or those of the publisher, the editors and the reviewers. Any product that may be evaluated in this article, or claim that may be made by its manufacturer, is not guaranteed or endorsed by the publisher.

## Supplementary material

Supplementary Figure 1 Biogenesis of exosomes. Exosomes are generated from late endosomes, which are formed by inward budding of the limited multivesicular body (MVB) membrane. Invagination of the late endosomes forms intraluminal vesicles (ILVs) within MVBs, where certain molecules of the parental cells such as proteins and nucleic acids are incorporated into ILVs. While some ILVs are trafficked to lysosomes for degradation, most fuse with cell membrane and are released into the extracellular space, forming ‘exosome’.

Supplementary Figure 2 Illustrative diagram on human immune system and its key players. Human immune system is composed of ‘innate immunity’ and ‘adaptive immunity’, where the latter is divided into ‘cell-mediated immunity’ and ‘humoral immunity’. Dendritic cells (DCs), macrophages and B cells are professional antigen presentation cells (APCs). During innate immune response, DCs can directly kill pathogens, infected or malignant cells, activate NK cells to aid in the killing process, and present foreign antigens to T cells to stimulate cell-mediated immune response; NK cells, once activated by DCs, can kill pathogens, infected or malignant cells directly, and activate macrophages; macrophages can directly kill pathogens, infected or malignant cells directly, and present foreign antigens to T cells. Innate immune response also involves other participants such as mast cells, basophils, eosinophils, neutrophils and complements. During cell-mediated immune response, T cells, on receival of antigens presented by DCs, macrophages and B cells, are amplified and kill infected or malignant cells by producing perforin (protein that forms pore on targeted cells). During humoral immunity, B cells recognize pathogens, amplify and secrete antibodies to kill them. On recognition of pathogens, infected or malignant cells, innate immune response is activated first (within 12h) followed by adaptive immune response (within days and can last weeks or even longer). Both innate and adaptive immune responses are firstly pro-inflammative (highest at 1-3h in innate immunity and 1-2d in adaptive immunity) and then anti-inflammative (highest at 6-12h in innate immunity and days in adaptive immunity).
