# Supplementary figures and images for: Emerging innovations on exosome-based onco-therapeutics
Source: Front Immunol. 2022 Aug 31;13:865245. doi: 10.3389/fimmu.2022.865245 (PMC9473149; doi:10.3389/fimmu.2022.865245)

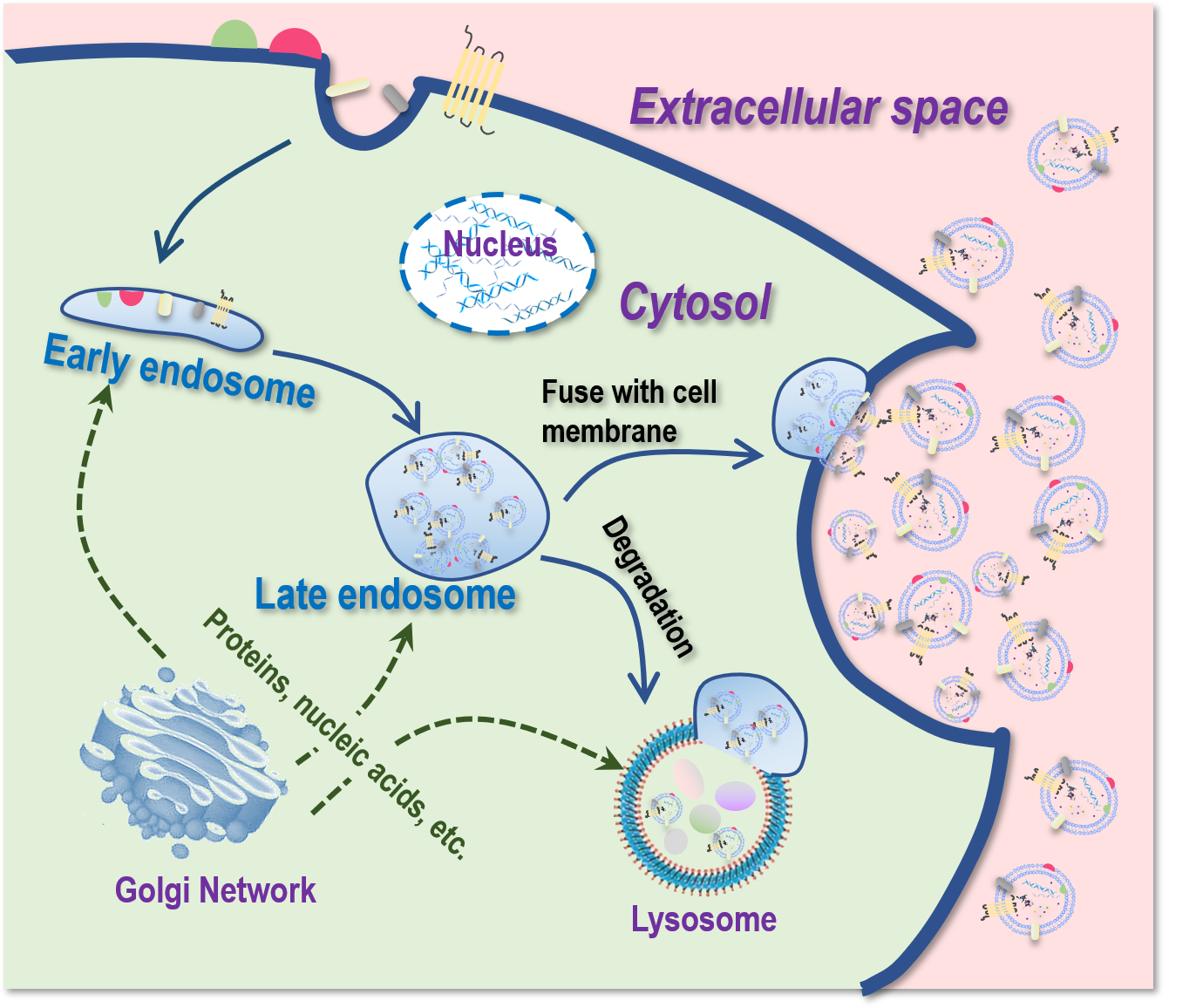

Supplement: Supplementary Figure 1 — Biogenesis of exosomes. Exosomes are generated from late endosomes, which are formed by inward budding of the limited multivesicular body (MVB) membrane. Invagination of the late endosomes forms intraluminal vesicles (ILVs) within MVBs, where certain molecules of the parental cells such as proteins and nucleic acids are incorporated into ILVs. While some ILVs are trafficked to lysosomes for degradation, most fuse with cell membrane and are released into the extracellular space, forming ‘exosome’. [file Image_1.tif]

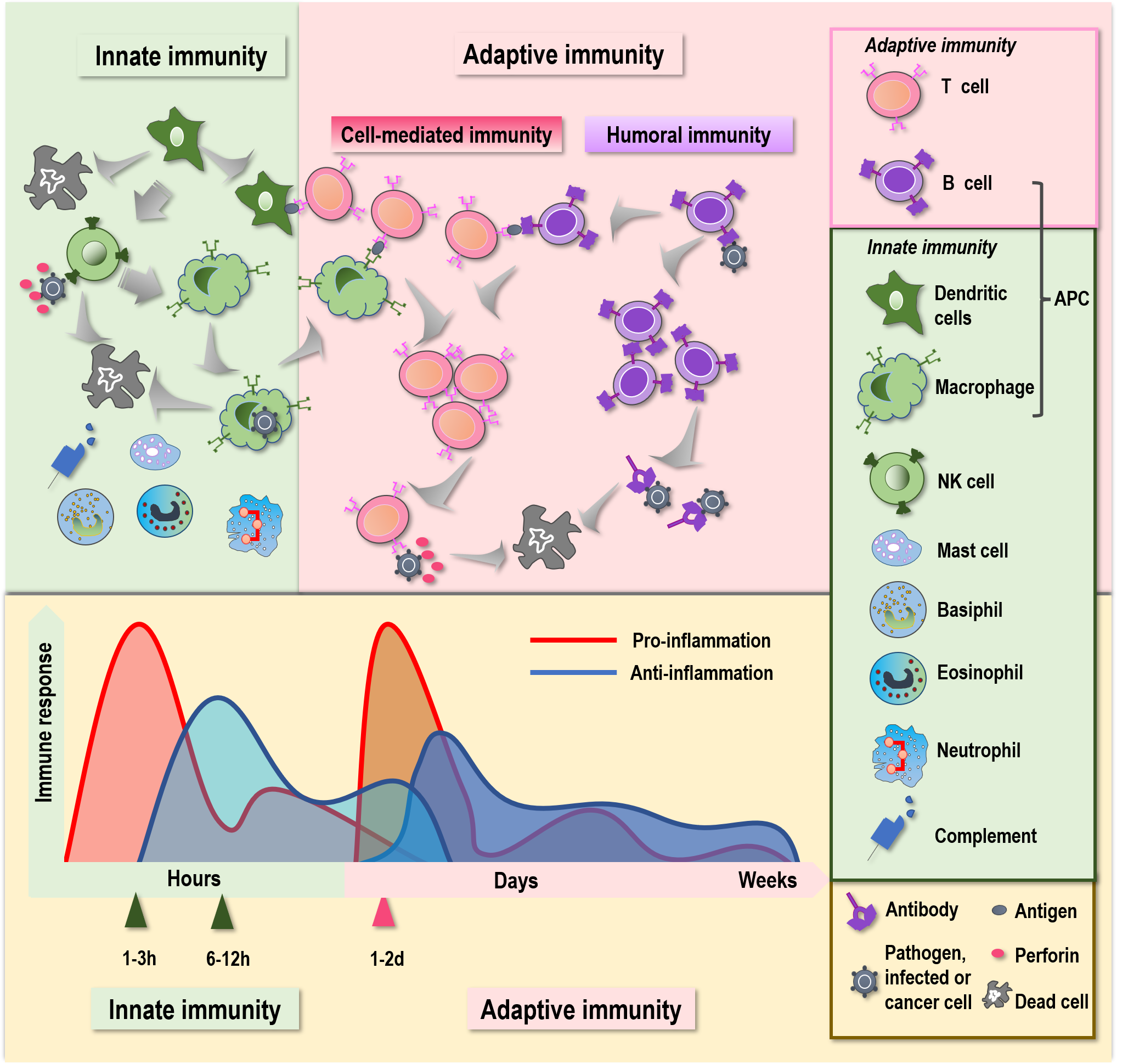

Supplement: Supplementary Figure 2 — Illustrative diagram on human immune system and its key players. Human immune system is composed of ‘innate immunity’ and ‘adaptive immunity’, where the latter is divided into ‘cell-mediated immunity’ and ‘humoral immunity’. Dendritic cells (DCs), macrophages and B cells are professional antigen presentation cells (APCs). During innate immune response, DCs can directly kill pathogens, infected or malignant cells, activate NK cells to aid in the killing process, and present foreign antigens to T cells to stimulate cell-mediated immune response; NK cells, once activated by DCs, can kill pathogens, infected or malignant cells directly, and activate macrophages; macrophages can directly kill pathogens, infected or malignant cells directly, and present foreign antigens to T cells. Innate immune response also involves other participants such as mast cells, basophils, eosinophils, neutrophils and complements. During cell-mediated immune response, T cells, on receival of antigens presented by DCs, macrophages and B cells, are amplified and kill infected or malignant cells by producing perforin (protein that forms pore on targeted cells). During humoral immunity, B cells recognize pathogens, amplify and secrete antibodies to kill them. On recognition of pathogens, infected or malignant cells, innate immune response is activated first (within 12h) followed by adaptive immune response (within days and can last weeks or even longer). Both innate and adaptive immune responses are firstly pro-inflammative (highest at 1-3h in innate immunity and 1-2d in adaptive immunity) and then anti-inflammative (highest at 6-12h in innate immunity and days in adaptive immunity). [file Image_2.tif]
